# Genetic transformation of cotton with a harpin-encoding gene *hpa*_*Xoo *_confers an enhanced defense response against different pathogens through a priming mechanism

**DOI:** 10.1186/1471-2229-10-67

**Published:** 2010-04-15

**Authors:** Weiguo Miao, Xiben Wang, Ming Li, Congfeng Song, Yu Wang, Dongwei Hu, Jinsheng Wang

**Affiliations:** 1 Department of Plant Pathology, Nanjing Agricultural University, Nanjing 210095, China; 2 College of Environment and Plant Protection, Hainan University, Haikou 570228, China; 3 Cereal Research Centre, Agriculture and Agri-Food Canada, Winnipeg, Manitoba, R3T 2N9, Canada; 4 Biotechnology Institute of Zhejiang University, Hangzhou 310029, China

## Abstract

**Background:**

The soil-borne fungal pathogen *Verticillium dahliae *Kleb causes *Verticillium *wilt in a wide range of crops including cotton (*Gossypium hirsutum*). To date, most upland cotton varieties are susceptible to *V. dahliae *and the breeding for cotton varieties with the resistance to *Verticillium *wilt has not been successful.

**Results:**

Hpa1_Xoo _is a harpin protein from *Xanthomonas oryzae *pv. *oryzae *which induces the hypersensitive cell death in plants. When *hpa1*_*Xoo *_was transformed into the susceptible cotton line Z35 through *Agrobacterium*-mediated transformation, the transgenic cotton line (T-34) with an improved resistance to *Verticillium dahliae *was obtained. Cells of the transgenic T-34, when mixed with the conidia suspension of *V. dahliae*, had a higher tolerance to *V. dahliae *compared to cells of untransformed Z35. Cells of T-34 were more viable 12 h after mixing with *V. dahliae *conidia suspension. Immunocytological analysis showed that Hpa1_Xoo_, expressed in T-34, accumulated as clustered particles along the cell walls of T-34. In response to the infection caused by *V. dahliae*, the microscopic cell death and the generation of reactive oxygen intermediates were observed in leaves of T-34 and these responses were absent in leaves of Z35 inoculated with *V. dahliae*. Quantitative RT-PCR analysis indicated that five defense-related genes, *ghAOX1, hin1, npr1, ghdhg-OMT*, and *hsr203J*, were up-regulated in T-34 inoculated with *V. dahliae*. The up-regulations of these defense-relate genes were not observed or in a less extent in leaves of Z-35 after the inoculation.

**Conclusions:**

Hpa1_Xoo _accumulates along the cell walls of the transgenic T-34, where it triggers the generation of H_2_O_2 _as an endogenous elicitor. T-34 is thus in a primed state, ready to protect the host from the pathogen. The results of this study suggest that the transformation of cotton with *hpa1*_*Xoo *_could be an effective approach for the development of cotton varieties with the improved resistance against soil-borne pathogens.

## Background

The soil-borne fungal pathogen *Verticillium dahliae *Kleb causes *Verticillium *wilt in a wide range of crops including cotton (*Gossypium hirsutum*). *V. dahliae *can be found in many cotton-growing areas and it has been considered as a major threat to the cotton production worldwide [1]. The reduction of cotton biomass caused by *Verticillium *wilt is mainly due to the discoloration of cotton leaves and stems vascular bundles, decreased photosynthesis, and increased respiration [2,3].

*V. dahliae *infects cotton roots and then grows into the host vascular system. Symptoms caused by *V. dahliae *in cotton include the necrosis on leaves, wilting, and the discoloration of vascular tissues. Plants infected with *V. dahliae *often develop characteristic mosaic patterns (leaves wilt with inter-veinal yellowing before becoming necrotic) [4]. Light to dark brown vascular discoloration is common in stems and branches of the infected cotton. Pathogenesis of *V. dahliae *is complicated due to the existence of defoliating and non-defoliating strains. The defoliating strains are the most virulent, which can cause typical symptoms of *Verticillium *wilt and lead to the complete defoliation of infected plants [1]. Cotton cultivars resistant to *Verticillium *wilt often show decreases in the rate of the disease progress and the symptom severity with a lower percentage of foliar symptoms [4]*Verticillium *wilt in cotton is usually controlled by cultural practices, such as the crop rotation [5], biological control with organic amendments [6], and fungicides [7]. Although the crop rotation and the application of organic amendments can be successfully in managing *Verticillium *wilt, these methods are not always practical [6]. Chemical fungicides are not environment-friendly and tend to raise concerns about the public health and the development of fungicide resistance in pathogens [8]. Moreover, none of the available commercial upland cotton varieties is immune to *V. dahliae *[9]. Conventional breeding methods for cotton varieties resistant to *Verticillium *wilt have not been successful.

Genetic engineering utilizing plant genes conferring disease resistance offers an alternative to conventional breeding methods for the improved resistance against pathogens, insects, or herbicides [10]. Genes encoding antifungal proteins, such as endochitinase [11], β-1,3-glucanases [12], and glucose oxidase [13], or components of signaling pathways involved in the defense response [14-17], have been used to generate transgenic plants resistant to various plant pathogens.

Several attempts have been made to generate transgenic cottons with a higher tolerance to *Verticillium *wilt. For example, a bean chitinase gene was transformed into cotton and crude leaf extracts from the transgenic cotton lines inhibited the growth of *V. dahliae in vitro *[18]. Furthermore, the transgenic cotton line with an over-expressed foreign *Gastrodia *anti-fungal protein was more resistant to *Verticillum *wilt than the untransformed cotton [19].

Harpins, encoded by *hrp *(hypersensitive response and pathogenicity) genes from Gram-negative plant pathogenic bacteria, are secreted through the Type III protein secretion systems (T3SSs) [20]. The T3SSs inject effector proteins directly into the cytosol of eukaryotic cells and allow the manipulation of host cellular activities to the benefit of the pathogen. In plant pathogenic bacteria, T3SSs are encoded by *hrp *(for hypersensitive response and pathogenicity) genes, which are capable of inducing host defense responses mediated by different signaling pathways, such as salicylic acid (SA) [21], jasmonic acid (JA) [21], and ethylene mediated pathways [22]. Harpin_Xoo _is a harpin-like protein encoded by *hpa1*_*Xoo *_derived from *Xanthomonas oryzae *pv. *oryzae *(Xoo), which belongs to *hpa *(hrp-associated) gene family related to the pathogenicity of *Xanthomonas *and the induction of hypersensitive response (HR) in non-host plants [23-27]. *Hpa1*_*Xoo *_encodes a 13.69 kDa glycine-rich protein with an amino acid composition similar to harpins from *Pseudomonas syringae *[28] and *Erwinia *species [29]. Hpa1_Xoo _also shares a high sequence similarity to PopA, a harpin-like protein, from *Ralstonia solanacearum *[30]. It has been proposed that Hpa could be involved in the secretion of Type III-dependent proteins. HpaA from *X. campestris *pv. *vesicatoria *promotes the secretion of pilus and effector proteins and therefore appears to be an important control protein of the T3SSs [31]. We had shown previously that the transformation of *hpa1*_*Xoo *_into tobacco conferred the improved resistance to *Alternaria alternata *and tobacco mosaic virus in the transgenic tobacco [32]. Similarly, a high level of resistance to all predominant races of *Magnaporthe grisea *in China was obtained in the rice line transformed with *hpa1*_*Xoo *_from *Xanthomonas oryzae *pv. *oryzae *[33]. Several other *hrp *genes have also been successfully transformed into different plant species including tobacco [32,34], potato [35], rice [33], and pear [36]. Unfortunately, the defense responses elicited by harpins and their active sites in hosts have not been fully understood.

In this study, a cotton transgenic line resistant to a range of soil-borne pathogens, including *V. dahliae*, was generated through the genetic transformation with *hpa1*_*Xoo *_from *Xoo*. The localization of hpa1_Xoo _in the transgenic cotton line was investigated. Furthermore the defense response and the expressions of defense-related genes in *hpa1*_*Xoo*_-expressing cotton line in response to *V. dahliae *were investigated.

## Results

### Generation of a *harpin_Xoo_*-transformed cotton line, namely T-34

Thirty transgenic T-34 plants and 5 untransformed Z35 plants were tested annually from 2003 to 2008. From T1 toT6, the transgenic cotton lines were screened for the resistance to kanamycin, the presence of *hpa1*_*Xoo *_insertion, and the expression of harpin_Xoo_. Only plants tested positive for these three attributes and showed an improved resistance to *Verticillium *wilt were selected and used for the further screening (see Additional file 1: Table S1). Resistance in the T6 progeny of T-34 line to *V. dahliae *segregated in a 3:1 ratio as a single Mendelian trait.

**Figure 1:**
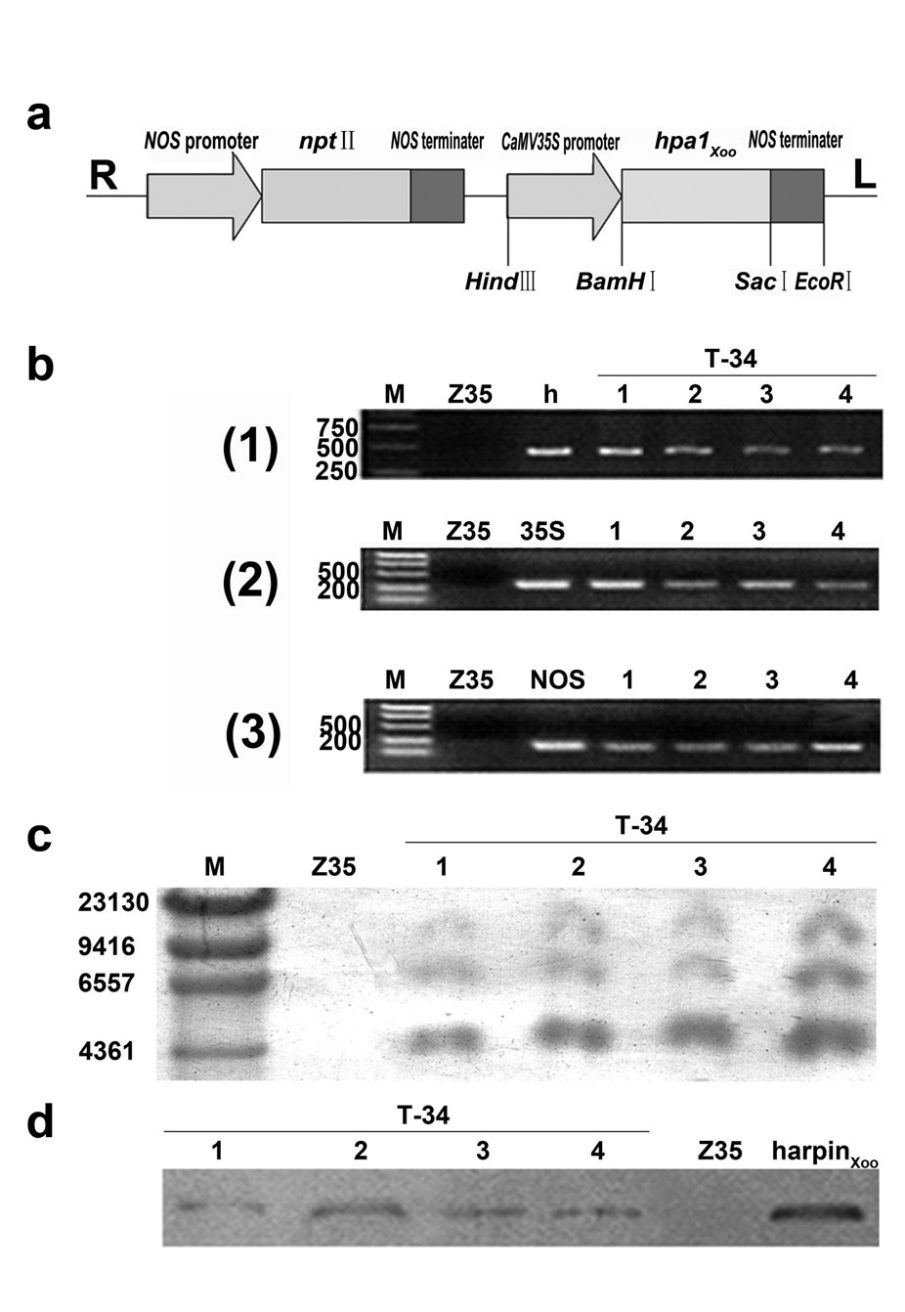
**Molecular analysis of *hpa1*_*Xoo*_-transformed T-34 and untransformed cotton**. (*Gossypium hirsutum*)**Z35**. **(a) **The schematic representation of recombinant plasmid pBI35S-*hpa1*_*Xoo*_-nptII. R and L represent the right and left borders of T-DNA. **(b) **Amplifications of *hpa1*_*Xoo *_(1), 35S promoter (2), and NOS terminator (3) in PCR; *hpa1*_*Xoo *_(h), 35S promoter (35S), and NOS terminator (NOS) represented the DNA fragment amplified from the positive control. M: marker; Four individual plants (1, 2, 3, 4) in T6 progeny of T-34 were tested. **(c) **Southern blot analysis of *hrp1*_*Xoo *_insertions in T-34 and Z35. Ten micrograms of genomic DNA was digested with *EcoRI *and hybridized against a DIG-labeled *hpa1*_*Xoo *_probe. M: marker. Four plants (1, 2, 3, 4) in T6 progeny of T-34 were tested. **(d) **Western blot analysis of harpin_Xoo _in T-34 transgenic lines. Four plants (1, 2, 3, 4) in T6 progeny of T-34 were tested. Purified harpin_Xoo _served as the positive control.

Four plants from T-34 line (T6 progeny) were randomly selected and used in the PCR analysis. Bands representing *hpa1*_*Xoo*_, 35S promoter, and NOS terminator (420 bp, 310 bp, and 180 bp, respectively) were detected in all four T-34 plants but they were absent in wild type Z35 plants (Figure 1a, b). Results of the PCR analysis were verified by the sequencing of amplification products and BLAST against appropriate sequences in the NCBI database http://www.ncbi.nlm.nih.gov (data not shown). The presence of *hpa1*_*Xoo *_inserts in transgenic plants was confirmed using Southern blot analysis against a DIG-labeled *hpa1*_*Xoo *_probe. Three bands, approximately 4.5 kb, 6.5 kb, and 10.5 kb in length, were detected using the DIG-labeled *hpa1*_*Xoo *_probe in the genomic DNA extracted from the four chosen T-34 plants. No positive signal was detected in untransformed Z35 (Figure 1c). The expression of harpin_Xoo _in cotton leaves was analyzed using a harpin_Xoo _polyclonal antibody. The band representing harpin_Xoo _was observed only in the total proteins extracted from leaves of *hpa1*_*Xoo*_-transformed T-34 (Figure 1d). All these results indicated that *hpa1*_*Xoo *_had been successfully transformed into T-34 and hpa1_Xoo _was constitutively expressed in the transgenic line T-34.

### *Verticillium *wilt resistance and the phenotype of transgenic T-34

The typical symptoms of *Verticillium *wilt first appeared on plants 10 days after the inoculation, and symptoms develop only when the temperature is below 30°C [1]. In our study, *Verticillium *wilt resistance of 45 *hpa1*_*Xoo*_-transformed T-34 plants inoculated with *V. dahliae *strains Vdps and V151 was assessed 10 days after the inoculation based on the degree of the foliar damage and vascular discoloration as described in the material and method. All plants were individually scored. The susceptible variety, Simian 3, and untransformed Z35 were used as the control. Ten days after the inoculation, only few chlorotic and necrotic spots were visible on leaves of T-34 whereas large chlorotic and necrotic areas were common in leaves of untransformed Z35 and the susceptible line Simian 3 (pictures not shown).

**Figure 2:**
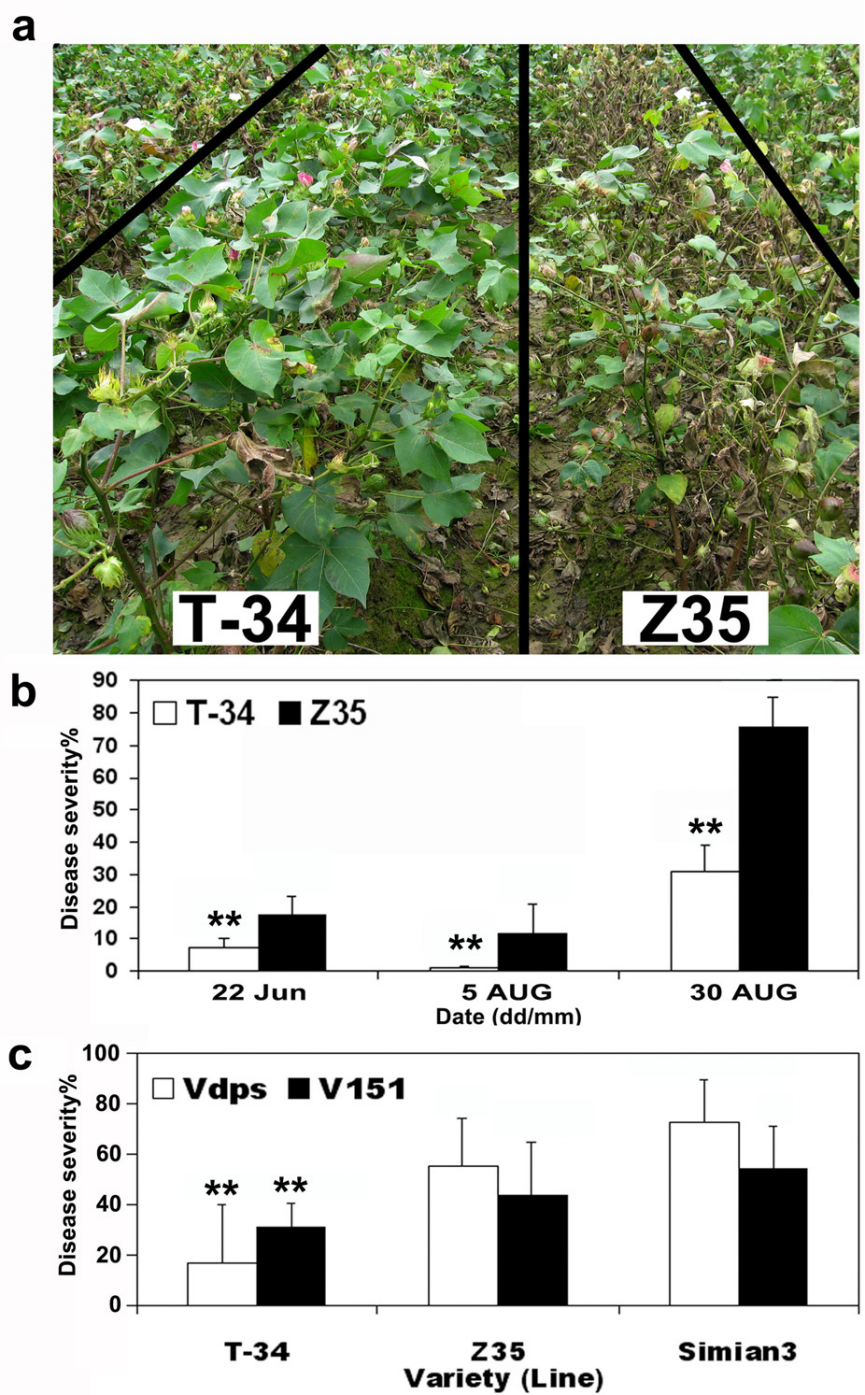
**Resistance of *hpa1*_*Xoo*_-transformed T-34 and untransformed Z35 to *Verticillium *wilt**. **(a) **Resistance phenotypes of *hpa1*_*Xoo*_-transformed T-34 and untransformed Z35 to *Verticillium *wilt in the nursery. **(b) **Disease severity of *Verticillium *wilt on *hpa1*_*Xoo*_-transformed T-34 and untransformed Z35 in the nursery. **(c) **Disease severity of *Verticillium *wilt on *hpa1*_*Xoo*_-transformed T-34 and untransformed Z35 in plastic pots. Average values and standard errors were calculated from 4 replicates. Simian 3 was the susceptible control. Asterisks represent significant differences at the level of 0.01.

The resistance of T-34 to *Verticillium *wilt was evaluated in field in 2008. A total of 200 plants were scored. The characteristic mosaic pattern of *Verticillium *wilt was rare in leaves of T-34 and no defoliation occurred during the growing season. In comparison, most Z35 plants showed severe *Verticillium *infections with the characteristic mosaic pattern on leaves and the defoliation occurred 2 or 3 months after the inoculation (Figure 2a).

The maximum temperature reached 32°C on August 5, 2008 and typical *Verticillium *symptoms were no longer visible. Disease assessment made from 22 June to 5 August showed that *Verticillium *wilt was significantly less severe in *hpa1*_*Xoo*_-transformed T-34, compared to untransformed Z35 and the susceptible variety Simian 3 (Figure 2b). The average *Verticillium *wilt ratings in *hpa1*_*Xoo*_-transformed T-34 were 7.32%-26.22% lower than those in untransformed Z35.

Although the defoliating strain V151 was more virulent than the non-defoliating strain Vdps [1] on T-34, the disease severity caused by these two stains were both lower on T-34, compared to the untransformed Z35 and the susceptible control Simian 3 (Figure 2c).

The transgenic T-34 and untransformed Z35 line shared similar phenotypic characteristics including the leaf morphology, and fiber quality (data not shown). Although the height of T-34 line was lower before the flowering stage, there was no significant difference between the height of T-34 and Z35 at and after the flowering stage (see Additional file 2: Figure S1).

### Localizations of harpin_Xoo _in transgenic cotton leaves and stem apices

**Figure 3:**
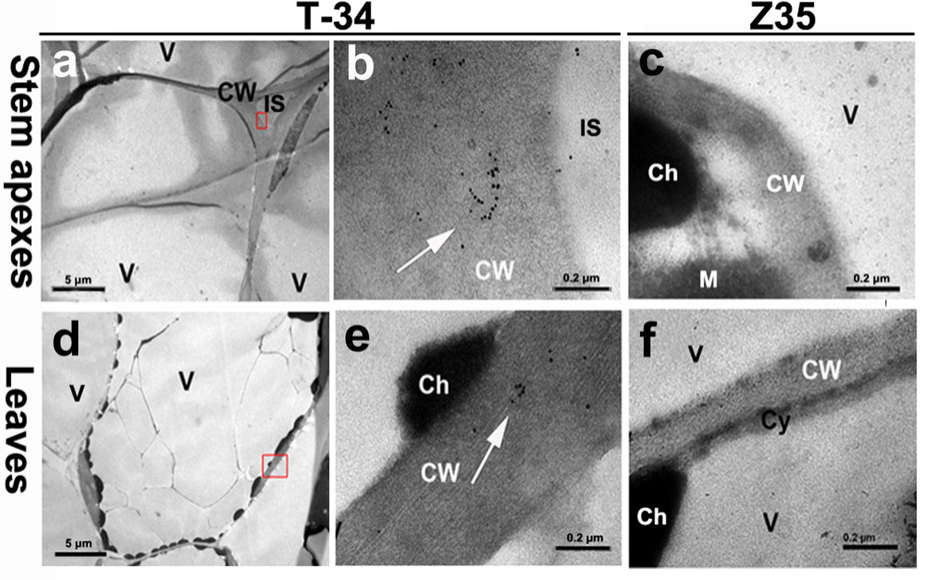
**Immuno-gold localization of harpin_Xoo _in leaves and stem apices of *hpa1*_*Xoo*_-transformed T-34 and untransformed Z35**. **(a) **and **(b) **Stem apices of *hpa1*_*Xoo*_-transformed T-34. **(c) **Stem apices of untransformed Z35. **(d) **and **(e) **Leaves of *hpa1*_*Xoo*_-transformed T-34. **(f) **Leaves of untransformed Z35. CW: cell wall, Cy: cytoplasm, V: vacuole, IS: intercellular space, Ch: chloroplasts, M: mitochondria. Bars: a and d = 5 μm; b, c, e, and f = 0.2 μm. Arrow points to gold particles labeled with harpin_Xoo _antiserum (15 nm particles). The squares indicate the regions of b and e magnified in a and d, respectively. More than 20 ultrathin sections of each sample were examined with a JEM × 1200 transmission electron microscope (Nikon, Japan). The experiment was repeated twice.

The localization of harpin_Xoo _in tissues of *hpa1*_*Xoo*_-transformed T-34 was investigated using the immuno-gold localization method. The harpin_*Xoo*_-labeled gold particles were not found in leaf and stem samples collected from the untransformed Z35 (Figure 3c and 3f) but they were clearly visible in leaf and stem samples from T-34 (Figure 3b and 3e). Harpin_Xoo_-labeled gold particles were mostly seen in clusters along the cell walls of leaves and in apical tissue of stems (Figure 3b and 3e). Each cluster contained an average of 10 to 20 gold particles. Only a few gold particles were found in cell membranes and chloroplasts. None was found in the mitochondria (Figure 3c).

### Oxidative burst in transgenic cotton T-34 triggered by inoculation

**Figure 4:**
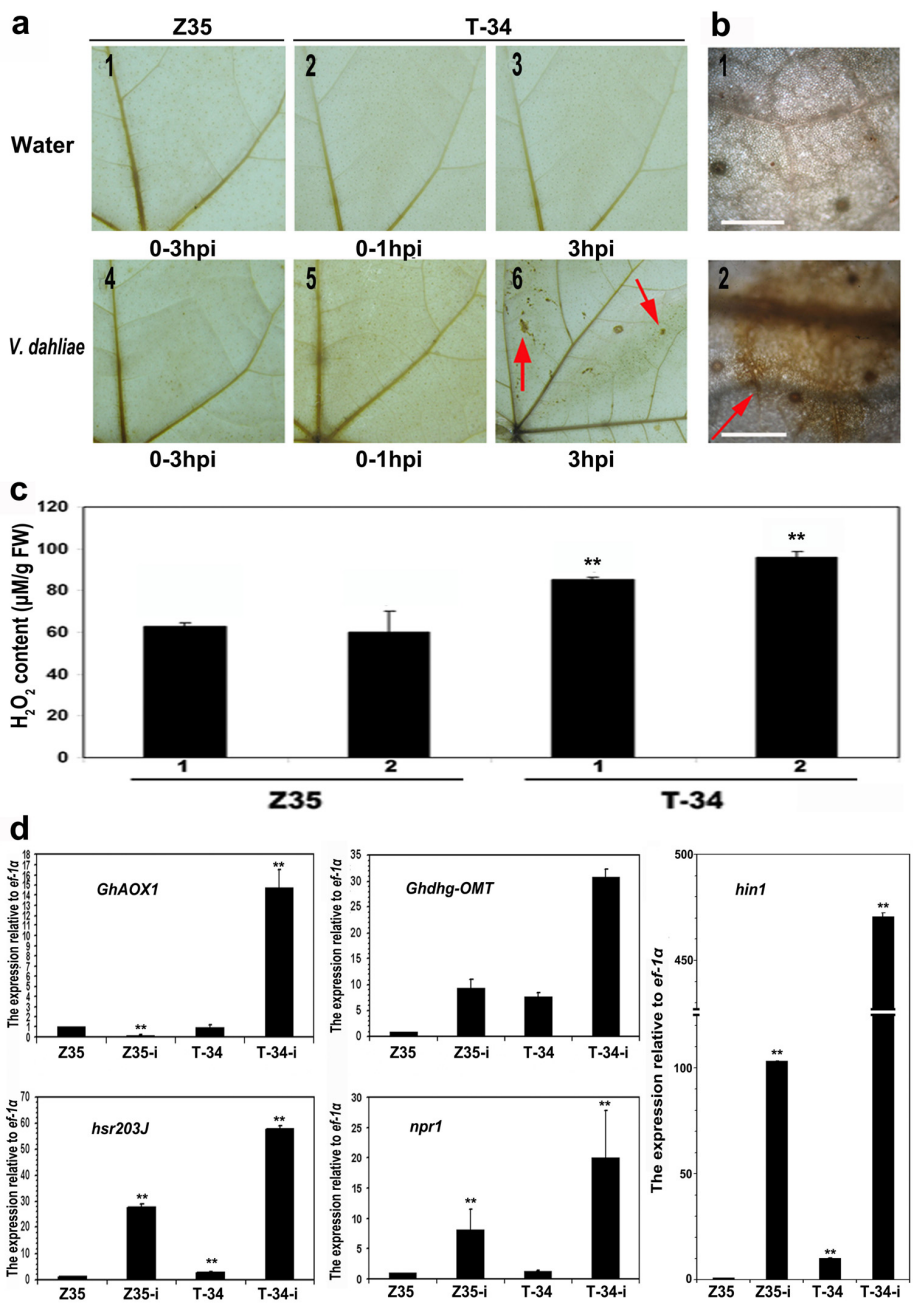
**Generation of active oxygen species (AOS) in leaves of *hpa1*_*Xoo*_-transformed T-34 and untransformed Z35**. **(a) **Oxygen burst in cotton leaves dipped in the conidial suspension of *Verticillium dahliae *collected from 0 to 3 hr after inoculation (red arrow points to the location of oxygen burst). **(b) **Light microscopy of the oxygen burst in leaves of untransformed Z35 (1) and *hpa1*_*Xoo*_-transformed T-34 (2) 3 h after the inoculation. **(c) **H_2_O_2 _content (μg/g fresh weight) in leaves of *hpa1*_*Xoo*_-transformed T-34 and untransformed Z35 dipped in the conidial suspension of *V*. *dahliae *(mean values and standard errors calculated from three replicates). 1, non-inoculated; 2, inoculated. **(d) **Quantitative RT-PCR analysis of *ghAOX1, hin1, npr1, ghdhg-OMT*, and *hsr203J *expression in leaves of *hpa1*_*Xoo*_-transformed T-34 (T-34-i) and untransformed Z35 (Z35-i) dipped in the conidial suspension of *V*. *dahliae *compared with that of *hpa1*_*Xoo*_-transformed T-34 (T-34) and untransformed Z35 (Z35) dipped in water (error bars indicate standard error). b (1, 2) scale bars = 0.01 mm. The experiment was repeated three times. Asterisks represent significant differences at the level of 0.01.

3, 3'-diaminobenzidine tetrahydrochloride (DAB) was used to detect the production of reactive oxygen intermediates (ROI) [37]. No reddish or brown spots representing the accumulation of H_2_O_2 _were observed in T-34 and Z35 leaves dipped in water. After the inoculation, visible reddish or brown spots were only observed in T-34 leaves collected 3 h after dipping in the conidial suspension of *V. dahliae *(Figure 4a, b). H_2_O_2 _content in T-34 and Z35 leaves dipped in the conidial suspension of *V*. *dahliae *was quantified using the method described by Jiang and Zhang (2001)[38]. The basal level of H_2_O_2 _was higher in leaves of transgenic T-34 than in leaves of Z35 prior to dipping. The level of H_2_O_2 _increased dramatically in leaves of transgenic T-34 3 h after dipping and such increase in H_2_O_2 _content was not observed in the treated Z35 leaves (Figure 4c).

The expressions of *ghAOX1 *[GenBank accession number DQ250028], *hsr203J *[GenBank accession number X77136], *hin1 *[GenBank accession number Y07563], and *npr1 *[39] were quantified using the real-time RT-PCR. *GhAOX1 *is a key gene involved in the production of active oxygen species (AOS) in plants [40,41] and *hsr203J *and *hin1 *are marker genes for HR which express specifically in plant tissues undergoing HRs [42,43]. The data was normalized to a constitutive expressed *ef-1α*. No up-regulations of *npr1, hsr203J, hin1 *and *ghdhg-OMT *were observed in the un-inoculated T-34 and Z-35 plants. The basal expression level of *GhAOX1 *was higher in the un-inoculated T-34, compared to that in wild type Z35. *Npr1, hsr203J, hin1 *and *GhAOX1 *were all up-regulated in T-34 and Z35 after plants were dipped in the conidial suspension of *V*. *dahliae*. Nevertheless, the up-regulations of these genes were stronger in leaves of transgenic T-34 in response to the dipping treatment (Figure 4d). In addition, the up-regulation of *dhg-OMT *[44] encoding hemigosspol was only observed in T-34 after the dipping treatment (Figure 4d).

### Microscopic hypersensitive response in transgenic T-34 after root inoculation with *Verticillium dahliae*

**Figure 5:**
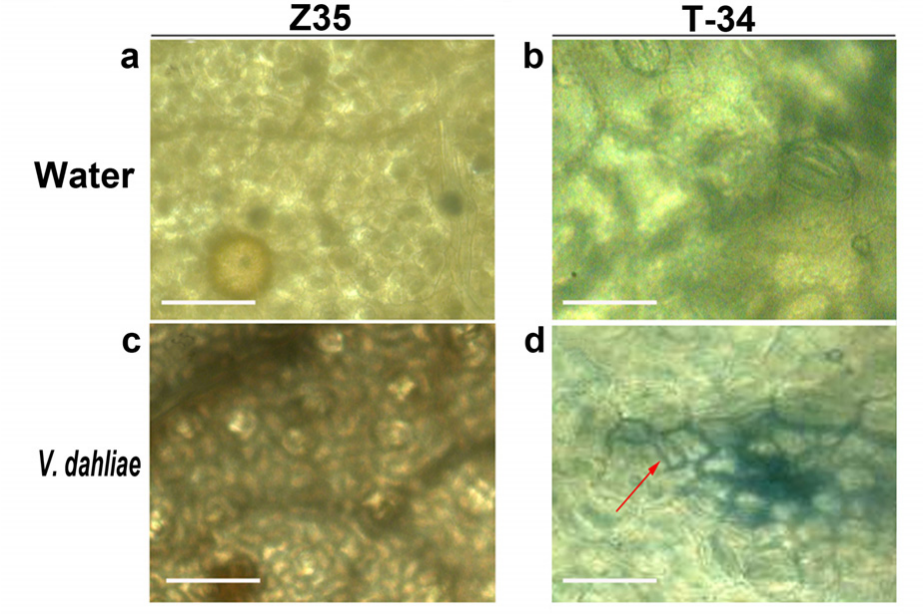
**Microscopic hypersensitive response (HR) in *hpa1*_*Xoo*_-transformed T-34 and untransformed Z35 20 days after root inoculations with *Verticillium dahliae***. **(a) **Leaves of uninoculated untransformed Z35. **(b) **Leaves of uninoculated *hpa1*_*Xoo*_-transformed T-34. **(c) **Leaves of untransformed Z35 inoculated with *V*. *dahliae*. **(d) **Leaves of *hpa1*_*Xoo*_-transformed T-34 inoculated with *V*. *dahliae *(red arrow indicates microscopic HR). (a), (b), (c), and (d) scale bars = 1 μm. The experiment was repeated three times.

Leaves were collected from T-34 and Z35 20 days after the root inoculation with *V. dahliae *conidia suspension in the green house and then stained with Trypan blue, which selectively stained dead or dying cells. Leaves inoculated with sterile water were used as the control. The results of microscopic examination were shown in Figure 5. No Trypan blue stained cells were observed in leaves of T-34 and Z35 treated with water (Figure 5a, b) and in leaves of Z35 inoculated with *V. dahliae *(Figure 5c). In comparison, large regions (1 to 5 μm^2^) of Trypan blue stained cells were observed in leaves of T-34 inoculated with *V. dahliae *indicating the occurrence of microscopic hypersensitive response (HR) (Figure 5d). The occurrence of regions of Tyrpan blue stained cells representing micro HRs (10-20 lesions per leaves) was observed in all leaves (≥ 4 leaves per plant) collected from 10 T-34 plants infected with *V. dahliae *(100%) whereas it was not observed in the controls (T-34 and Z35 un-inoculated) (0%).

### Tolerance of *harpin_Xoo_*-transformed T-34 cells in suspension to *Verticillium dahliae*

**Figure 6:**
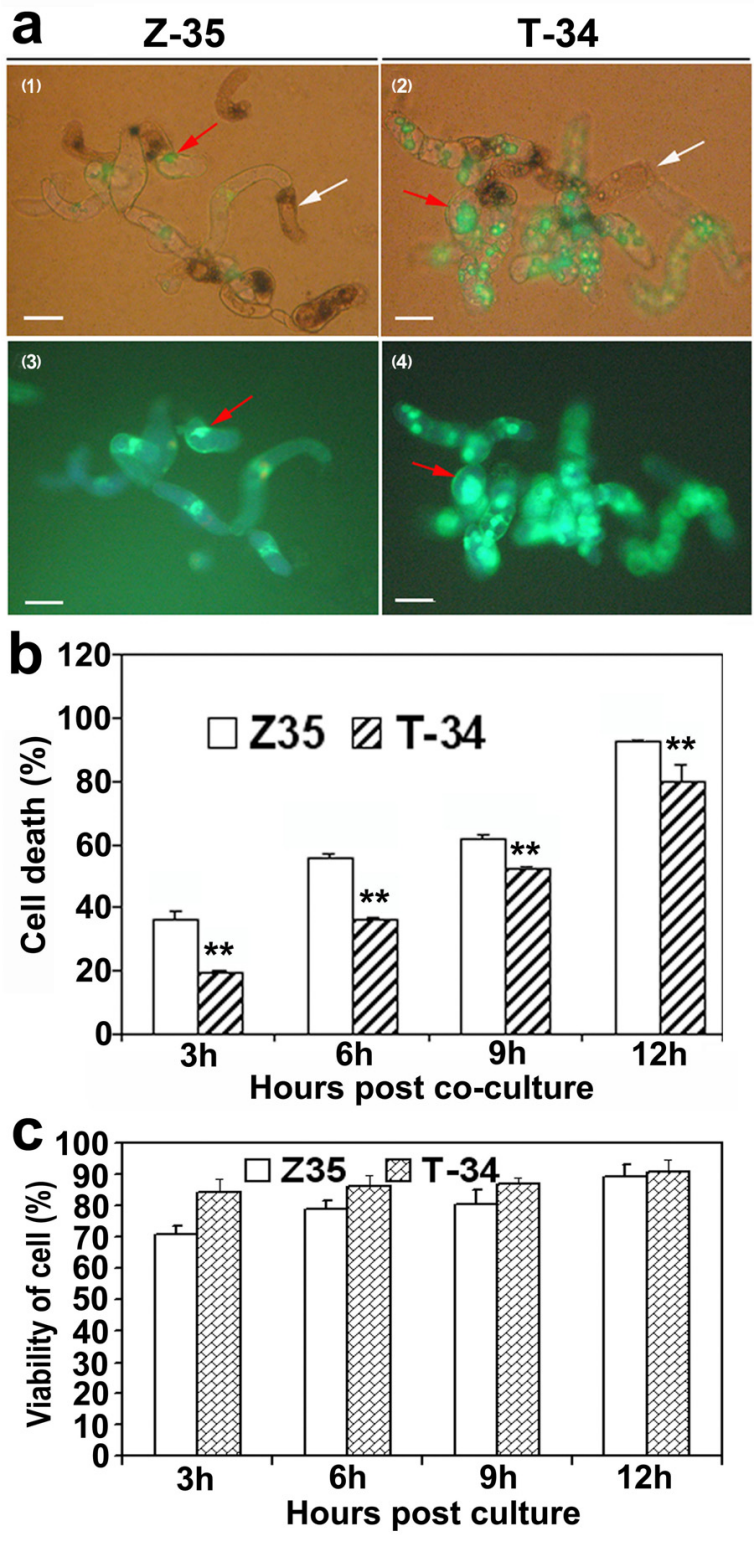
**Viability of cotton cells in the presence of conidia of *Verticillium dahliae***. **(a) **(1) Living (red arrow) or dead (white arrow) cells in the cell suspension of untransformed Z35 mixed with conidia of *V*. *dahliae *under a conventional light microscope. (2) Living (red arrow) or dead (white arrow) cells in the cell suspension of *hpa1*_*Xoo*_-transformed T-34 mixed with conidia of *V*. *dahliae *under a conventional light microscope. (3) Fluorescence emitted from living cells (red arrow) of untransformed Z35 mixed with conidia of *V*. *dahliae *under a fluorescence microscope. (4) Fluorescence emitted from living cells (red arrow) of *hpa1*_*Xoo*_-transformed T-34 mixed with conidia of *V*. *dahliae *under a fluorescence microscope. (1), (2), (3), and (4) scale bars = 300 μm. **(b) **Percentage of the cell death in the cotton cell suspension mixed with *V. dahliae *conidia. **(c) **Percentage of cotton cells in the absence of conidia of *Verticillium dahliae*. Cotton cells and *V*. *dahliae *conidia were mixed in a ratio of 1:20. The percentage of cell death was counted at 3, 6, 9, and 12 h after mixing. Error bars indicate standard error of the mean (n = 3). Data points marked with asterisks are significantly different (Student's *t *test, *p *< 0.01). The experiment was repeated three times.

To determine the reaction of *harpin*_*Xoo*_-transformed cotton cells to *V. dahliae*, cell suspensions of *harpin*_*Xoo*_-transformed T-34 and untransformed Z35 were mixed with the conidial suspension of *V. dahliae *in a ratio of 1: 20 by volume. The viability of cotton cells was counted at 3, 6, 9, and 12 h after mixing with *V. dahliae *conidial suspension under a fluorescence microscope by staining with fluorescein diacetate (FDA). Fluorescence emitted from T-34 cells was stronger than that from Z35 cells (Figure 6a). The percentage of cell death in T-34 cell suspension mixed with *V. dahliae *conidia was significantly lower than that in Z35 cell suspension mixed with *V. dahliae *from 3 to 12 h (Figure 6b). The viability of Z35 and T34 cells was similar in the absence of *V. dahliae *conidia and almost 100% of untreated Z35 and T34 cells were viable after 12 h (Figure 6c).

## Discussion

In our previous study, we reported that harpin_Xoo_, applied as a foliar spray, conferred cotton the resistance to *Verticillium *wilt in a *Verticillium *cotton nursery [45]. In this study, *hpa1*_*Xoo *_was transformed into a susceptible upland cotton variety Z35. During the screening process, the *hpa1*_*Xoo*_-transformed cotton lines were more resistant not only to *Verticillium *wilt but also to *Fusarium *wilt caused by *Fusarium oxysporum *f. sp. *vasinfectum *(see Additional file 3: Figure S2). The non-specific resistance is often related to the up-regulation of *npr1*. NPR1 is thought to be a key transcriptional regulator in plant defense responses involving multiple signaling pathways [32]. In this study, the up-regulation of *npr1 *was observed in *hpa1*_*Xoo *_transformed T-34 after the inoculation with *V. dahliae*. This could indicate that the improved resistance in cotton mediated by the transformation of *hpa1*_*Xoo *_is likely to be non-specific. In addition, cells of the transformed T-34 plants were more tolerant to *V. dahliae*, compared to cells of Z35, when they were cultured with *V. dahliae *conidia suspension (Figure 6). It is possible that the improved resistance in *hpa1*_*Xoo*_-transformed cotton plants is also related to the improved tolerance of cotton cells to *V. dahliae*.

It should be noted that the *hpa1*_*Xoo*_-transformed cotton was not entirely immune to *V. dahliae *under our test condition. Similarly several harpin expressing transgenic plants only showed enhanced, but not complete, resistance to a wide range of pathogens [32-34]. Since harpins often act as effectors which induce systemic acquired resistance rather than the immunity in plants, these results are not surprising. Secondly, Z35 is a very susceptible variety. The highly virulent, defoliating strains were dominant in the area where transgenic plants were tested. The inoculum level of *V. dahliae *in the region was extremely high due to the recent *Verticillum *wilt outbreak [46]. It is possible that the incomplete resistance of *hpa1*_*Xoo*_-transformed cotton against *V. dahliae *is partially due to the high level of inoculum and the aggressive *V. dahliae *pathotype in the region [1].

To date, the action site of harpins in plants remains unknown. An early study of *harpinEa *and *harpinPss *indicated that the plant cell wall was critical for HR inducing activity of *harpinEa *and *harpinPss *[47]. Tampakaki and Panopoulos [48] suggested that the receptor(s) for harpin could be extracellular in transgenic tobacco transformed with *hrpZPsph*. More recently, immuno-cytological analyses showed that HR phenotype of transgenic tobacco was related to the presence of PopA at the plasma membrane, which was involved in the formation of an ion-conducting channel allowing the passage of true effectors into plant cells [49,50]. This discrepancy indicates that the binding sites of harpins in plants vary depending on their origins. In our study, *harpin*_*Xoo *_was detected as clustered particles mainly along the cell walls of transformed T-34. This result was in an agreement with that reported by Hoyos *et al*. [47] and indicated that the cotton cell wall could be important for the HR inducing activity of *harpin*_*Xoo *_in the transgenic cotton. Similarly, Sohn *et al*. (2007) [34] reported that the action site of harpins located in the plant cell walls. It remains not clear that how Harpins were secreted to cotton cell walls in the transgenic plants since the signal peptide was not included in *harpin*_*Xoo *_used for the trasnformation. Similarly, several previous studies showed that transformation of harpin-encoding genes without known signal peptides into rice and tobacco resulted in the *in vivo *expression of harpins, which conferred the improved resistance against different pathogens [32-34]. The secretion of Harpins to the plant cell wall in the *harpin*_*Xoo *_transformed cotton suggests the presence of unknown signal peptide in *harpin*_*Xoo *_which is recognized by cotton. It is also possible that *harpin*_*Xoo *_may utilize the plant signal peptide during its *in vivo *expression. Although the even distribution of immuno-gold labeled particle is normal in the immunocytological study [51-53], the distribution of immuno-gold particles in clusters is not uncommon [54,55].

It has been reported that the defense responses induced by harpins were different between the endogenous and exogenous applications. For example, visible HRs, accompanied by the up-regulation of HR marker genes, often occur in tobacco leaves infiltrated with Harpins [21]. Only microscopic HRs can be observed when Harpin is sprayed onto leaves despite the similar up-regulation of HR marker genes in treated plants [56]. In transgenic plants expressing Harpin, the defense responses are more complicated in response to the pathogen infection. The transgenic plants show a stronger response to the pathogen infection resulting from the substantial increase in the expression of the defense related genes, such as the marker genes for HR and SAR and those encoding anti-microbial proteins [32-34].

The defense response and transcriptional expression of multi-defense genes were significantly enhanced in the *harpin*_*Xoo *_transformed T-34 compared to that seen in untransformed Z35. In addition, we also compared the transcriptional difference in a genomic wide analysis between *hpa1*_*Xoo*_-transformed T-34 and untransformed Z35 through microarray analysis in which over 1000 genes involved in 162 pathways were found to be regulated differently (unpublished data). These results suggest an altered regulation of genes involved not only in the disease resistance but also in many metabolic pathways in the *harpin*_*Xoo *_transformed plants. This unique physiological condition is very similar to the so-called primed state [57]. The primed plants often display faster and/or stronger activation of cellular defenses to various stresses and depend on the key regulator of induced resistance, namely *npr1*. Over the past decade, the priming of defensive responses in plants by pathogen-associated molecular patterns (PAMP; elicitor) triggered by plant pathogens has been increasingly evident [57-60].

In our study, *npr1 *was slightly more up-regulated in the transgenic T-34 compared to that in wild type Z35 in response to *V. dahliae*. Similarly the up-regulation of *npr1 *was also observed in the transgenic *hpa1*_*Xoo *_tobacco but it was not found in the transgenic *hpaGEP *tobacco in response to the pathogen infection [32,34]. This difference in the expression of *npr-1 *in different transgenic plants expressing Harpins could be due to the differences either in the receptor of the target gene or in the sites of their insertions in the plant genome.

In leaves of transgenic T-34, micro HR occurred in response to the inoculation of *V. dahliae*. In addition, the more rapid accumulation of H_2_O_2 _and up-regulation of *ghAOX1 *and *hsr203J *were observed in T-34, compare to those in wild type Z35, after the inoculation. Harpin can induce HR which is associated with the generation of reactive oxygen intermediates as a proximal response. A rapid burst of reactive oxygen species (ROS) followed by a chain of events often occur in plants treated with harpins [21,56]. It is still questionable whether the micro HR observed in the transgenic T-34 is directly related to the infection caused by *V. dahliae *since no *V. dahliae *was observed in the sites of micro HRs. It is possible that such micro HR could augment the defense response in the transformed T-34 by the gentle PCD (programmed cell death) correlated with the enhanced expression of HR marker genes. Micro HR could contribute to the resistance against *Verticillium *wilt through a priming mechanism. The primed state also explains the higher basal levels of H_2_O_2 _in leaves of the transgenic line (T-34). *Dhg-OMT *was also up-regulated in T-34 after inoculations with *V. dahliae*. Since *dhg-OMT *encodes one key enzyme in the biosynthesis of terpenoids in cotton. It indicates that the phytoalexin-like compound may be also involved in the defense response of cotton against *V. dahliae *[44].

## Conclusions

Hpa1_Xoo _accumulates along the cell walls of the transgenic T34, where it could trigger the generation of H_2_O_2 _as a cell wall endogenous elicitor. T-34 is thus in a primed state, ready to protect the hosts from pathogens. Multiple defense responses are induced in the transgenic T-34 in response to the infection caused by *V. dahliae*. *Hin1 *(ndr1) and *hsr203j *are up-regulated in T-34 indicating that the genes related to HR are activated without any visible HR phenotype in the transgenic plants.

## Methods

### Plant transformation

ZhongMian 35 (Z35) (*Gossypium hirsutum *L.) was used to generate transgenic cotton lines expressing *harpin*_*Xoo *_using an *Agrobacterium tumefaciens*-mediated method described by Shao et al. (2008) [33]. A pGEM-T vector containing *hpa1*_*Xoo *_(pGEM-*hpa1*_*Xoo*_) was digested with BamH1 and Sac1. The BamH1- and Sac1-digested *hpa1*_*Xoo *_fragment was ligated into a pBI121 vector (Clontech, Palo Alto, CA, USA) to generate the recombinant binary vector pBI35S-*hpa1*_*Xoo*_-nptII, which contained a neomycin phosphotransferase II (nptII) with a nopaline synthase (nos) promoter and terminator, a CaMV35S promoter, an *hpa1*_*Xoo *_insert, and a nopaline synthase terminator (Figure 1a). The binary vector pBI35S-*hpa1*_*Xoo*_-nptII was mobilized into *Agrobacterium tumefaciens*-disarmed helper strain LBA4404 by the heat shock method [61]. Hypocotyl segments of Z35 were used as explants for the transformation, and the transformants were selected using the method described by Sunilkumar and Rathore (2001) [62].

Kanamycin resistance tests, PCR analysis, and Southern and Western Blot were used to screen T1 to T6 progeny for transgenic *harpin*_*Xoo *_cotton lines with desirable phenotypes including improved resistance to *Verticillium *wilt and fiber quality. Only T6 progeny from transgenic line T-34 were used in this study and untransformed Z35 (receptor) was used as the negative control. Cultivated cotton cultivar Simian 3 was used as the susceptible control for evaluating resistance to *Verticillium *wilt in the study.

### Fungal materials

A non-defoliating *V. dahliae *strain Vdps and a defoliating *V. dahliae *strain V151, obtained from Dr. Ling Lin at the Jiangsu Academy of Agricultural Science (China), were used in our the study. *V*. *dahliae *strains were maintained on potato dextrose agar (PDA) at 25°C. For the preparation of the inoculum, PDA plates were flooded with a conidial suspension of *V. dahliae *and the flooded plates were incubated at 25°C for 7 days. The PDA plates were then flooded with 50 ml sterile distilled water to collect the conidia using the method described by Joost *et al*. (1995) [63]. The conidia were washed once with 100 ml sterile distilled water and the suspension was diluted to a concentration of 1-3 × 10^7 ^conidia/ml. The conidial suspension of *V. dahliae *strain Vdps was used to inoculate the roots of cotton plants and for other experiments.

### Evaluation of resistance to *Verticillium dahliae*

After the surface disinfection for 5 min with a 5% solution of sodium hypochlorite, cotton seeds were sown in a potting mixture (mould and sand, 6:1, v/v). Fifteen 2-week-old cotton seedlings were carefully uprooted and the roots were immersed for 15 min in 100 ml of conidial suspension containing 1-3 × 10^7 ^conidia per ml. Fifteen control plants were immersed in sterile distilled water. All plants were then replanted in a plastic pot (9 cm in diameter) and grown under 12 h of light at 25°C and 70%-90% relative humidity.

Pathogenicity was determined based on both external (foliar damage) and internal (vascular discoloration) symptoms 10 and 20 days after inoculation, respectively. Foliar damage was evaluated by rating the symptom on the cotyledon and leaf of inoculated plant (X) according to the following rating scale: 0 = no foliar symptoms; 1 = yellowing or necrosis of 1-2 cotyledons; 2 = cotyledon fall or yellowing of a leaf; 3 = more than 2 wilted or necrotic leaves; 4 = dead leaf. Foliar alteration index (FAI) was calculated for each inoculated plant: FAI = 100∑X/(4n), where (4) is the maximum score for each plant (maximum score for each plant = 4), (n) the total number of inoculated plant. Vascular discoloration was evaluated according to the method described by Yang et al. (2008) [64]; discoloration was scored (y) for every internode using the following scale: 0 = no discoloration; 1 = less than 25% localized brown regions within the vascular tissue of the same internode; 2 = 25%-70% localized brown regions within the vascular tissue of the same internode; 3 = more than 70% browning of vessels but not of the adjacent tissues; 4 = browning of both vessels and adjacent tissues. The browning Index (BI) was calculated as follows; BI = 100∑y/4d; where (d) is the total number of seedling internodes including hypocotyls and (4) is the maximum score for an internode. Mean values of FAI and/or BI as Disease severity (DS) were calculated based on four replicates for both inoculated and control plants.

In 2008, the resistance of transgenic cotton was evaluated in a naturally infested *Verticillium *wilt nursery in DaFeng city, Jiangsu province, China. The soil in the nursery is sandy-loam with pH of 8.5. Except for the higher temperature (>30°C) in August, average temperature at the nursery usually ranges between 20°C and 25°C during the growing season, which is conducive for the development of *Verticillium *wilt.

Seeds of the transgenic cotton were sown in the field in early May of 2008. Irrigation was provided as needed during the growing season. The experimental plot was divided into four subplots. Each subplot consisted of two rows. Each row is 5 m long and 4 m wide and comprises 15 plants spaced 0.3 m apart. Each treatment was replicated four times, and the replicates were arranged in a randomized complete block design. Untransformed cotton Z35 served as the negative control for *Verticillium *wilt. The testing materials in each replicate were sown randomly in each subplot. The trial plot was separated by at least 50 m from other breeding materials of cotton and sprayed with pesticides to control insect pests. Scoring for disease severity started after the first symptoms appeared on leaves; subsequently, the scoring was conducted on 22 June, 5 August, and 30 August 2008.

### Kanamycin resistance tests

Seedlings were screened for kanamycin resistance (Amresco, Solon., Ohio, USA) at the 3- to 4- leaf stage. Kanamycin was applied onto the leaf surface at a concentration of 5000 mg/L. kanamycin-susceptible seedlings, which changed from green to yellow, were discarded a week after the Kanamycin treatment. The treatment was repeated three times and only kanamycin-resistant plants were retained for further study.

### DNA extraction, PCR analysis, and Southern blot analysis

Total genomic DNA was extracted from leaves of transgenic cotton line T-34 and the untransformed Z35, using a AxyPrep Multisource Genomic DNA Miniprep Kit (Axygen Biosciences, California, USA). Primers for *hpa1*_*Xoo*_, CaMV35S promoter, and NOS terminator (listed in Table 1) were used in the PCR assays. PCR reactions were carried out in a 25 μl reaction volume containing 1× PCR buffer (Applied Biosystem), 1.5 mM MgCl_2_, 0.2 mM dNTPs, 2.5 mM forward and reverse primers, 0.5 U Taq polymerase, and 30 ng sample DNA. Amplifications were performed in a thermal cycler (GeneAmp PCR 9700) using the following temperature profile: initial denaturation at 95°C for 2 min; 35 cycles at 95°C for 30 s, 60°C for 30 s, and 72°C for 1 min; and a final extension at 72°C.

**Table 1:** Oligonucleotides used in PCR and quantitative RT-PCR

Gene	Primer sequence	Anneal temperature (°C)	Segment length (bp)
hpa1Xoo(EF028092*)	forward:5'-TTCGGATCCATGAACTCTTTGAACACACAATT-3' reverse:5'-GGTGAGCTCTTACTGCATCGATGCGCT-3'	56	438
35S	forward:5'-AGAGGCTTACGCAGCAGGTC-3' reverse:5'-GCCAGTCTTTACGGCGAGTT-3'	52	310
NOS	forward:5'-GAACTGACAGAACCGCAACG-3' reverse:5'-ACCGAGGGGAATTTATGGAA-3'	50	180
GhAOX 1(DQ250028)	forward:5'-GCGCCTGGGGATGATGATGAGTCGTG-3' reverse:5'-GCGCTTCAGTGATAACCGAGCGGAG-3'	57	1298
hsr203J(X77136)	forward:5'-TGTACTACACTGTCTACACGC-3' reverse:5'-GATAAAAGCTATGTCCCACTCC-3'	55	618
EF-1α (AJ223969)	forward:5'-AGACCACCAAGTACTACTGCAC-3' reverse:5'-CCACCAATCTTGTACACATCC-3'	58	495
Ghdhg-OMT(GQ303569)	forward:5'-ATGAATATGGGCAATGCTAAT-3' reverse:5'-TCAGGGGTAAACCTCAATGAGA-3'	53	1087
npr1(U76707)	forword:5'-GGCCTCGAGATGGCTTATTTGTCTGAGCCATCATCT-3' reverse:5'-CGTCTCGAGTCACAATTTCCTATACTTGTAGG-3'	62	1794
hin1(Y07563)	forword:5'-GAACGGAGCCTATTATGGCCCTTCC-3' reverse:5'-CATGTATATCAATGAACACTAAACGCCGG-3'	55	867

* GenBank accession numbers

For the Southern blot analysis, 3 μg genomic DNA extracted from leaves of the transgenic T-34 and untransformed Z35 was digested with restriction endonuclease EcoR1 (TaKaRa Biotechnology (Dalian) Co. Ltd, China) in a final volume of 50 μl. The digested genomic DNA was separated on 1.5% (w/v) agarose gel and then transferred onto a hybond- N+ nylon membrane (Roche Applied Science, Mannheim, Germany) after denaturation using the method prescribed by the manufacturer. The probe for hybridizations was amplified from an *hpa1*_*Xoo *_fragment and then labeled with digoxigenin using DIG-High Prime DNA Labeling Kit (Roche Applied Science, Mannheim, Germany). The hybridization signal was detected using a DIG-High Prime DNA Detection Kit (Roche Applied Science, Mannheim, Germany).

### Western blot analysis

Total proteins were extracted from leaves of transgenic T-34 and untransformed Z35 according to the manufacturer's instructions for P-CelLytics Plant Cell Protein Extraction Kit (Shenergy Biocolor Bioscience and Technology Co., Shanghai, China). Total proteins were separated on a 15% sodium dodecyl sulfate-polyacrylamide gel electrophoresis (SDS-PAGE) and then transferred onto a polyvinylidene fluoride (PVDF) membrane (Roche Applied Science, Mannheim, Germany). The membranes were blotted with a polyclonal antibody developed against *harpin*_*Xoo *_and goat anti-rabbit IgG-HRP antibody (Sino-American Biotech, Luoyang, China). The color was developed using DAB.

### Preparation of plant samples and immuno-gold labeling

Samples were collected from the second and the third fresh leaves and stem apex of four T-34 and two Z35 plants at 4 to 5 leaf stage. The leaf samples were first fixed in a mixture of 3% (v/v) paraformaldehyde and 1% glutaraldehyde in 50 mmol phosphate-buffered saline (PBS), pH 7.2, at 4°C for 3 h. The samples were then washed with the same buffer and dehydrated in 50% ethanol at 4°C for 1 h, followed by washings with 50%, 70%, 90%, and 100% ethanol (3 times each) at -20°C for 2 h. Finally, the samples were embedded in K4M resin and polymerized under UV array at -20°C for 3 days and incubated at the room temperature for 2 days. Ultrathin sections were cut with a diamond knife and collected on Formvar-coated nickel grids.

Colloidal gold particles, 15 nm in diameter, were prepared as described by Slot and Geuze (1985) [65] and coated with Protein A at pH 6.0. Harpin_Xoo _antiserum was used for the localization of harpin_Xoo _and the immuno-labeling was performed at 28°C. The ultrathin sections were floated on a drop of double-distilled water for 5 min; the samples were then transferred to the blocking solution (BL) and incubated for 60 min. Harpin_Xoo _antiserum was added to the BL in a dilution of 1:200; the samples were incubated for 60 min; floated first on BL for 60 min and then on PA-gold for 120 min. The samples were thoroughly washed with double-distilled water 3 times and air-dried. More than 20 ultrathin sections of each sample were examined with a JEM ×1200 transmission electron microscope (Nikon, Japan). The experiment was repeated twice.

### Preparations of cotton cell suspension and assay for tolerance to *Verticillium dahliae*

Suspensions of Z35 and T-34 cells were prepared using the method described by Wu et al. (2005) [66]. The cells were cultured in a 500 ml round-bottom flask containing 200 ml modified MS medium (MS; 2,4-D 0.1 mg/L, kinetin 0.1 mg/L, maltose 30 g/L; pH 5.8). The flasks were placed under illumination for 12 h with continuous shaking (120 rpm). Cells were sub-cultured weekly by a 4-fold dilution until harvest. The suspensions of cotton cells and of the conidia were then mixed in a ratio of 1:20. The final concentration of both the cells and the conidia in the mix was the same, namely 1-3 × 10^7 ^cells/mL. Death of cotton cells in the mixture was quantified using the FDA stain method described by Amano et al. (2003) [67]. The number of viable cells emitting fluorescence was counted under a fluorescence microscope (Olympus, Japan). The percentage of dead cells was calculated as [(total cells - cells emitting fluorescence)/total cells] × 100. The experiment was repeated three times.

### Microscopy for micro hypersensitive response

Roots of transgenic T-34 and untransformed Z35 plants at the 2 to 3 leaf stage were inoculated with a conidial suspension of *V. dahliae *(1-3 × 10^7^/ml) according to the method described above. Leaves were collected 10 days after the inoculation and stained with Trypan blue using the method described by Lipka et al. (2005) [68]. Stained leaf samples were observed under a Leica light microscope (Leica DMRB, Leica Microsystems, Germany) and photographed with a Leica DFC camera (DM2500-3HF-FL, Leica Microsystems, Germany). Leaves (≥ 4 leaves per plant) without any wound or visible symptom of the disease from 10 independent T-34 plants were examined.

### Observation of oxidative burst and quantification of H_2_O_2 _in cotton leaves

The second and third cotton leaves with freshly cut petioles and no visible wounds were collected when the plants were at the 5 leaf stage. Two-third of the petiole was immersed into 10 ml of conidial suspension (1-3 × 10^7 ^ml) for 0, 1, or 3 h. To make the accumulation of H_2_O_2 _in the cotton leaves visible, fresh inoculated leaf samples were incubated in 1 mg/ml of DAB (pH 3.8) for 8 h and then decolorized in 96% ethanol. The samples were mounted on slides with 60% glycerol and examined under an Olympus light microscope (BH-2). The accumulation of H_2_O_2 _was visible as a reddish or brown discoloration. Furthermore, the production of H_2_O_2 _in leaves was quantified. Leaves dipped in sterile water were used as the negative control. The production of H_2_O_2 _in leaves was measured 0, 1, and 3 h after inoculation with a commercial H_2_O_2 _detection kit (Nanjing Jiancheng Bioengineering Institute, Nanjing, China) using the method described by Jiang and Zhang (2001) [38] and expressed as a percentage of fresh weight. The experiment was repeated three times.

### Quantitative RT-PCR

The second and third fully grown leaves of cotton were harvested when the plants were at the 5 leaf stage and inoculated as described above. Leaves treated with sterile distilled water served as control. The leaf blades were frozen in liquid nitrogen immediately after the sampling. Total RNA was extracted from the leaves using a commercial kit, namely RNAiso, from TaKaRa Biotechnology Co. Ltd, Dalian, China. RNA concentrations were quantified using a biophotometer (Eppendorf AG, Hamburg, Germany). cDNA was prepared with a TaKaRa PrimeScript RT-PCR kit (TaKaRa Biotechnology Co. Ltd, Dalian, China). Two-step qRT-PCR was performed on an ABI PRISM 7000 (ABI, Foster City, CA, USA) using a SYBR Premix EX TaqTM kit (TaKaRa Biotechnology Co. Ltd, Dalian, China). All PCR reactions were repeated three times and the data were normalized to constitutively expressed ef-1α using the 2-ΔΔCT method described by Livak and Schmittgen (2001) [69]. The primer sequences used in the quantitative RT-PCR are listed in Table 1.

### Statistical analysis

For quantitative determination, the data were analyzed by the *t *test at *P *= 0.05 or 0.01 using the Microsoft Analysis Tool. For differences in disease severity, each transgenic plant was compared with an untransformed plant.

## Authors' contributions

The studies were conceived and planned by JSW. WGM, XBW and ML carried out the molecular genetic studies, participated in the sequence alignment and drafted the manuscript. CFS and YW participated in the sequence alignment, the design of the study, and performed the statistical analysis. DWH carried out the immunoassays. The manuscript was edited and prepared by JSW along with WGM and XBW. All authors read and approved the final manuscript.

## Supplementary Material

Additional file 1 **Table S1**. **Stability of resistance to *Verticillium dahliae *in T1-T6 progenies of transgenic cotton line T-34**. ^+^: a score of 0-4 was given based on both external (foliar damage) and internal (vascular discoloration) symptoms 10 and 20 days after inoculation, respectively. Plants showed the ratings of 0 - 2 were counted as resistant (R) and those with the ratings of 3-4 were counted as susceptible (S). ^++^: +/- represented the presence/absence of the amplification product using *hpa1*_*Xoo *_specific primers in the PCR analysis. Click here for file

Additional file 2 **Figure S1**. **The plant height of *hpa1*_*Xoo*_-transformed T-34 and untransformed Z35 at different growing stages**. The experiment with three replications was performed at an independent field in Dafeng city, Jiangsu, CHINA. The fertilizer, irrigation, plant protection and other inter cultural practices were according to normal agronomic practices. The height of continued fifty plants was investigated each replication at same time. Click here for file

Additional file 3 **Figure S2**. **Isolate F24 of *Fusarium oxysporum *f. sp. *vasinfectum *(FOV) race 7 was used in this study**. Inoculum was prepared by autoclaving cotton seed (at 121°C and 103.4 kPa for 20 min) twice and mixing it with monoconidial cultures of Fov that had been grown on PDA. When fully colonized (10 days), the inoculum was mixed with pasteurized UC potting mix (sorghum: potting mix, 1:10 v/v) in plastic bags and incubated for 4 weeks. The colonized cotton seed-UC mix was then added to more pasteurized potting mix (1:1, v/v) and distributed equally into pots 9 cm in diameter. The transgenic *hpa1*_*Xoo *_cotton line T-34, the receptor Z35, and the susceptible cotton cultivar Simian 3 were grown from seed in the potting mix containing the inoculum. One plant of each cultivar was grown in each pot and there were 10 replications (pots) of each treatment (isolate of Fov). The experiments were repeated three times. All plants were grown under 12 h of light at 24-29°C and 70%-90% relative humidity. Individual plants were rated for disease severity based on the following scale for vascular discoloration. The discoloration was scored (y) for every internode. 0 = no vascular staining evident, 1 = light vascular staining evident as spotty areas, 2 = more contiguous staining covering an area equal to between one-quarter and one-half of the transverse section of the stem, 3 = moderate vascular staining (intensity of the brown/black color) evident as a band extending over nearly all of the transverse section, 4 = vascular staining darker or the plant dead. The disease index (DI) was calculated as follows: DI = 100∑y/4d, where (d) is the total number of seedling internodes including hypocotyls and (4) is the maximum score for an internode. Mean values of DI were calculated based on four replicates for both inoculated and control plants. Asterisks represent significant differences at the level of 0.01. Click here for file

## References

[B1] Bell AA Hillocks RJ Verticillium wilt Cotton Diseases 1992 C.A.B. International, Wallingford, U.K. 87 126

[B2] Hampton RE Wullschleger SD Oosterhuis DM Impact of Verticillium wilt on net photosynthesis, respiration and photorespiration in field-grown cotton (*Gossypium hirsutum *L.) Physiological and Molecular Plant Pathology 1990 37 271 280 10.1016/0885-5765(90)90076-A

[B3] Paplomatas EJ Bassett DM Broome JC DeVay JE Incidence of Verticillium wilt and yield losses of cotton cultivars (*Gossypium hirsutum*) based on soil inoculum density of *Verticillium dahliae* Phytopathology 1992 82 1417 1420 10.1094/Phyto-82-1417

[B4] Pegg GF Tjamos EC, Beckman C Pathogenesis in vascular disease of plants Vascular wilt diseases of plants 1989 Springer, Berlin, Germany 51 94

[B5] Xiao CL Subbarao KV Schulbach KF Koike ST Effects of crop rotation and irrigation on *Verticillium dahliae *microsclerotia in soil and wilt in cauliflower Phytopathology 1998 88 1046 1055 10.1094/PHYTO.1998.88.10.1046 18944816

[B6] Huang J Li H Yuan H Effect of organic amendments on Verticillium wilt of cotton Crop Protection 2006 25 1167 1173 10.1016/j.cropro.2006.02.014

[B7] Gray FA Koch DW Influence of late season harvesting, fall grazing, and fungicide treatment on Verticillium wilt incidence, plant density, and forage yield of alfalfa Plant Disease 2004 88 811 816 10.1094/PDIS.2004.88.8.811 30812507

[B8] Kurt S Dervis S Sahinler S Sensitivity of *Verticillium dahliae *to prochloraz and prochloraz-manganese complex and control of Verticillium wilt of cotton in the field Crop Protection 2003 22 51 55 10.1016/S0261-2194(02)00097-2

[B9] Colson-Hanks ES Deverall BJ Effect of 2,6-dichloroisonicotinic acid, its formulation materials and benzothiadiazole on systemic resistance to alternaria leaf spot in cotton Plant Pathology 2000 49 171 178 10.1046/j.1365-3059.2000.00439.x

[B10] Punja ZK Genetic engineering of plants to enhance resistance to fungal pathogens: a review of progress and future prospects Canadian Journal of Plant Pathology 2001 23 216 235

[B11] Gentile A Deng Z La-Malfa S Distefano G Domina F Vitale A Polizzi G Lorito M Tribulato E Enhanced resistance to *Phoma tracheiphila *and *Botrytis cinerea *in transgenic lemon plants expressing a *Trichoderma harzianum *chitinase gene Plant Breeding 2007 126 146 151 10.1111/j.1439-0523.2007.01297.x

[B12] Wrobel-Kwiatkowska Lorenc-Kukula MK Starzycki M Oszmianski J Kepczynska E Szopa J Expression of beta-1,3-glucanase in flax causes increased resistance to fungi Physiological and Molecular Plant Pathology 2004 65 245 256 10.1016/j.pmpp.2005.02.008

[B13] Lee YH Yoon IS Suh SC Kim HI Enhanced disease resistance in transgenic cabbage and tobacco expressing a glucose oxidase gene from *Aspergillus niger* Plant Cell Reports 2002 20 857 863 10.1007/s00299-001-0416-x

[B14] Komatsu K Okuda S Takahashi M Matsunaga R Nakazawa Y QTL mapping of pubescence density and flowering time of insect-resistant soybean Genetics and Molecular Biology 2007 30 635 639 10.1590/S1415-47572007000400022

[B15] Yang KY Kim YM Lee SH Song PS Soh MS Overexpression of a mutant basic helix-loop-helix protein HFR1, HFR1-deltaN105, activates a branch pathway of light signaling in Arabidopsis Plant Physiology 2003 133 1630 1642 10.1104/pp.103.029751 14645731 PMC300719

[B16] Cao H Li X Dong X Generation of broad-spectrum disease resistance by overexpression of an essential regulatory gene in systemic acquired resistance Proceedings of the National Academy of Sciences of the United States of America 1998 95 6531 6536 10.1073/pnas.95.11.6531 9601001 PMC34547

[B17] Chern M Fitzgerald HA Canlas PE Navarre DA Ronald PC Overexpression of a Rice NPR1 Homolog Leads to Constitutive Activation of Defense Response and Hypersensitivity to Light Molecular Plant-Microbe Interactions 2005 18 511 520 10.1094/MPMI-18-0511 15986920

[B18] Tohidfar M Mohammadi M Ghareyazie B Agrobacterium-mediated transformation of cotton (*Gossypium hirsutum*) using a heterologous bean chitinase gene Plant Cell Tissue and Organ Culture 2005 83 83 96 10.1007/s11240-004-6155-2

[B19] Wang YQ Chen DJ Wang DM Huang QS Yao ZP Liu FJ Wei XW Li RJ Zhang ZN Sun YR Over-expression of Gastrodia anti-fungal protein enhances Verticillium wilt resistance in coloured cotton Plant Breeding 2004 123 454 459 10.1111/j.1439-0523.2004.01005.x

[B20] Alfano JR Collmer A The type III (Hrp) secretion pathway of plant pathogenic bacteria: trafficking harpins, Avr proteins, and death Journal of Bacteriology 1997 179 5655 5662 9294418 10.1128/jb.179.18.5655-5662.1997 PMC179450

[B21] Dong H Delaney TP Bauer DW Beer SV Harpin induces disease resistance in Arabidopsis through the systemic acquired resistance pathway mediated by salicylic acid and the NIM1 gene Plant Journal 1999 20 207 215 10.1046/j.1365-313x.1999.00595.x 10571880

[B22] Dong HP Peng J Bao Z Meng X Bonasera JM Chen G Beer SV Dong H Downstream divergence of the ethylene signaling pathway for harpin-stimulated Arabidopsis growth and insect defense Plant Physiology 2004 136 3628 2638 10.1104/pp.104.048900 15516507 PMC527161

[B23] Li P Long JY Huang YC Zhang Y Wang JS A novel member of avrBs3 gene family from *Xanthomonas oryzae *pv. *oryzae *has a dual function Process in Natural Sciences 2004 14 767 773

[B24] Zou LF Wang XP Xiang Y Zhang B Li YR Xiao YL Wang JS Walmsley AR Chen GY Elucidation of the *hrp *Clusters of *Xanthomonas oryzae *pv. *oryzicola *that control the hypersensitive response in nonhost tobacco and pathogenicity in susceptible host Rice Applied and Environment Microbiology 2006 72 6212 6224 10.1128/AEM.00511-06 PMC1563621 16957248

[B25] Gough CL Genin S Lopes V Boucher CA Homology between the HrpO protein of *Pseudomonas solanacearum *and bacterial proteins implicated in a signal peptide-independent secretion mechanism Molecular and General Genetics: MGG 1993 239 378 392 10.1007/BF00276936 8316211

[B26] Huguet E Hahn K Wengelnik K Bonas U hpaA mutants of *Xanthomonas campestris *pv. *vesicatoria *are affected in pathogenicity but retain the ability to induce host-specific hypersensitive reaction Molecular Microbiology 1998 29 1379 1390 10.1046/j.1365-2958.1998.01019.x 9781876

[B27] Zhu W Magbanua MM White FF Identification of two novel *hrp*-associated genes in the *hrp *gene cluster of *Xanthomonas oryzae *pv. *oryzae* Journal of bacteriology 2000 182 1844 1853 10.1128/JB.182.7.1844-1853.2000 10714988 PMC101866

[B28] He SY Huang HC Collmer A *Pseudomonas syringae *pv. *syringae *harpinPss: A protein that is secreted via the *hrp *pathway and elicits the hypersensitive response in plants Cell 1993 73 1255 1266 10.1016/0092-8674(93)90354-S 8324821

[B29] Gaudriault S Brisset MN Barny MA HrpW of *Erwinia amylovora*, a new Hrp-secreted protein FEBS letters 1998 428 224 228 10.1016/S0014-5793(98)00534-1 9654138

[B30] Arlat M Gijsegem FV Huet JC Pernollet JC Boucher CA PopA1, a protein which induces a hypersensitivity-like response on specific *Petunia *genotypes, is secreted via the Hrp pathway of *Pseudomonas solanacearum* EMBO Journal 1994 13 543 553 8313899 10.1002/j.1460-2075.1994.tb06292.x PMC394843

[B31] Lorenz C Kirchner O Egler M Stuttmann J Bonas U Büttner D HpaA from Xanthomonas is a regulator of type III secretion Molecular Microbiology 2008 69 344 360 10.1111/j.1365-2958.2008.06280.x 18485076

[B32] Peng JL Bao ZL Ren HY Wang JS Dong HS Expression of Harpin_Xoo _in transgenic tobacco induces pathogen defense in the absence of hypersensitive cell death Phytopathology 2004 94 1048 1055 10.1094/PHYTO.2004.94.10.1048 18943792

[B33] Shao M Wang JS Dean RA Lin YG Gao XW Hu SJ Expression of a harpin-encoding gene in rice confers durable nonspecific resistance to *Magnaporthe grisea* Plant Biotechnology Journal 2008 6 73 81 18005094 10.1111/j.1467-7652.2007.00304.x

[B34] Sohn S Kim Y Kim B Lee S Lim CK Hur JH Lee J Transgenic tobacco expressing the hrpNEP Gene from *Erwinia pyrifoliae *triggers defense responses against *Botrytis cinerea* Molecular Cells 2007 24 232 239 17978576

[B35] Li R Fan Y Reduction of lesion growth rate of late blight plant disease in transgenic potato expressing harpin protein Science in China (Ser. C) 1999 42 96 101 10.1007/BF02881754 18726504

[B36] Malnoy M Venisse JS Chevreau E Expression of a bacterial effector, harpin N, causes increased resistance to fire blight in *Pyrus communis* Tree Genet Genomes 2005 1 41 49 10.1007/s11295-005-0006-0

[B37] Thordal-Christensen H Zhang Z Wei Y Collinge DB Subcellular localization of H_2_O_2 _in plants. H_2_O_2 _accumulation in papillae and hypersensitive response during the barley powdery mildew interaction Plant Journal 1997 11 1187 1194 10.1046/j.1365-313X.1997.11061187.x

[B38] Jiang M Zhang J Effect of abscisic acid on active oxygen species, antioxidative defence system and oxidative damage in leaves of maize seedlings Plant and Cell Physiology 2001 42 1265 1273 10.1093/pcp/pce162 11726712

[B39] Wang X Dou D Wang Z Jia S Cloning full-length cDNA of GbNPR1 gene from *Gossypium barbadense *and its expression in transgenic tobacco Scientia Agricultura Sinica 2006 39 886 894

[B40] Li F Zhang Y Wang M Zhang Y Wu X Guo X Molecular cloning and expression characteristics of alternative oxidase gene of cotton (*Gossypium hirsutum*) Molecular Biology Reports 2008 35 97 105 10.1007/s11033-007-9058-6 17351819

[B41] Maxwell DP Wang Y McIntosh L The alternative oxidase lowers mitochondrial reactive oxygen production in plant cells Proceedings of the National Academy of Sciences of the United States of America 1999 96 8271 8276 10.1073/pnas.96.14.8271 10393984 PMC22224

[B42] Pontier D Godiard L Marco Y Roby D Hsr203J, a tobacco gene whose activation is rapid, highly localized and specific for incompatible plant Plant Journal 1994 5 507 521 10.1046/j.1365-313X.1994.5040507.x 8012404

[B43] Pontier D Balague C Bezombes MI Tronchet M Deslandes L Roby D Identification of a novel pathogen-responsive element in the promoter of the tobacco gene *HSR203J*, a molecular marker of the hypersensitive response Plant Journal 2001 26 495 507 10.1046/j.1365-313x.2001.01049.x 11439136

[B44] Liu J Benedict CR Stipanovic RD Magill CW Bell AA Cloning and expression of desoxyhemigossypol-6-O-methyltransferase from cotton (*Gossypium barbadense*) Journal of Agricultural and Food Chemistry 2002 50 3165 3172 10.1021/jf011701y 12009981

[B45] Miao W Wang L Zhang S Nurzya Sheng M Jin W Tan Z Adjustment and control of two biocontrol products with *hrp *gene against cotton Verticillium wilt Xinjiang Agricultural Sciences 2004 41 299 302

[B46] Chen R Wang K The population genetics of cotton Verticillium Wilt in China Cotton Science 2001 13 209 212

[B47] Hoyos AE Stanley CM He SY Pike S Pu XA Novacky A The interaction of harpin_Pss _with plant cell walls Molecular Plant-Microbe Interactions 1996 9 608 616

[B48] Tampakaki AP Panopoulos NJ Elicitation of hypersensitive cell death by extracellularly targeted HrpZPsph produced in planta Molecular Plant-Microbe Interactions 2000 13 1366 1374 10.1094/MPMI.2000.13.12.1366 11106029

[B49] Racapé J Belbahri L Engelhardt S Lacombe B Lee J Lochman J Arai AS Nicole M Nürnberger T Parlange F Puverel S Keller H Ca^2+ ^dependent lipid binding and membrane integration of PopA, a harpin-like elicitor of the hypersensitive response in tobacco Molecular Microbiology 2005 58 1406 1420 16313625 10.1111/j.1365-2958.2004.04910.x

[B50] Lee J Klusener B Tsiamis G Stevens C Neyt C Tampakaki AP Panopoulos NJ Nöller J Weiler EW Cornelis GR Mansfield JW Nürnberger T HrpZ (Psph) from the plant pathogen *Pseudomonas syringae *pv. *phaseolicola *binds to lipid bilayers and forms an ion conducting pore in vitro Proceedings of the National Academy of Sciences of the United States of America 2001 98 294 10.1073/pnas.011265298 PMC14583 11134504

[B51] Chevalier J Kazatchkine MD Distribution in clusters of complement receptor type one (CR1) on human erythrocytes The Journal of Immunology 1989 142 2031 2036 2522131

[B52] Colitti M Musetti R Stefanon B Detection of apoptosis-inducing factor in involuting mammary tissue by immunoelectron microscopy Micron 2004 35 307 310 10.1016/j.micron.2003.08.002 15003619

[B53] Evergren E Tomilin N Vasylieva E Sergeeva V Bloom O Gad H Capani F Shupliakov O A pre-embedding immunogold approach for detection of synaptic endocytic proteins in situ Journal of Neuroscience Methods 2004 135 169 174 10.1016/j.jneumeth.2003.12.010 15020101

[B54] Fahimi HD Reich D Völkl A Baumgart E Contributions of the immunogold technique to investigation of the biology of peroxisomes Histochemistry and Cell Biology 1996 106 105 114 10.1007/BF02473205 8858370

[B55] Fournier J Françoise E Villemeur T Robain O Corinne I Deslys J Dormont D Brown P Distribution and submicroscopic immunogold localization of cellular prion protein (PrPc) in extracerebral tissues Cell and Tissue Research 1998 292 77 84 10.1007/s004410051036 9506914

[B56] Dayakar BV Lin HJ Chen CH Ger MJ Lee BH Pai CH Chow D Huang HE Hwang SY Chung MC Feng TY Ferredoxin from sweet pepper (*Capsicum annuum *L.) intensifying harpinPss-mediated hypersensitive response shows an enhanced production of active oxygen species (AOS) Plant Molecular Biology 2003 51 913 924 10.1023/A:1023061303755 12777051

[B57] Conrath U Beckers GJM Flors V Garcia-Agustin P Jakab G Mauch F Newman MA Pieterse CM Poinssot JB Pozo MJ Pugin A Schaffrath U Ton J Wendehenne D Zimmerli L Mauch-Mani B Priming: Getting ready for battle Molecular Plant-Microbe Interactions 2006 19 1062 1071 10.1094/MPMI-19-1062 17022170

[B58] Durrant WE Dong X Systemic acquired resistance Annual Review of Phytopathology 2004 42 185 209 10.1146/annurev.phyto.42.040803.140421 15283665

[B59] Goellner K Conrath U Priming: it's all the world to induced disease resistance European Journal of Plant Pathology 2008 121 233 242 10.1007/s10658-007-9251-4

[B60] Trouvelot S Varnier AL Allegre M Mercier L Baillieul F Arnould C Gianinazzi Pearson V Klarzynski O Joubert JM Pugin A A o-1,3 glucan sulfate induces resistance in grapevine against *Plasmopara viticola *through priming of defense responses, including HR-like cell death Molecular Plant-Microbe Interactions 2008 21 232 243 10.1094/MPMI-21-2-0232 18184067

[B61] An G Ebert P Mitra A Ha S Gelvin SB, Schilperoort RA Binary vectors Plant Molecular Biology Manual 1988 Dordrecht, Netherlands: Kluwer Academic Publishers 1 19

[B62] Sunilkumar G Rathore KS Transgenic cotton: factors influencing Agrobacterium-mediated transformation and regeneration Molecular Breeding 2001 8 37 52 10.1023/A:1011906701925

[B63] Joost O Bianchini G Bell AA Benedict CR Magill CW Differential induction of 3-hydroxy-3-methylglutaryl CoA reductase in two cotton species following inoculation with *Verticillium* Molecular Plant-Microbe Interactions 1995 8 880 885 8664497 10.1094/mpmi-8-0880

[B64] Yang C Guo WZ Li GY Gao F Lin SS Zhang TZ QTLs mapping for Verticillium wilt resistance at seedling and maturity stages in *Gossypium barbadense *L Plant Science 2008 174 290 298 10.1016/j.plantsci.2007.11.016

[B65] Slot JW Geuze HJ A new method of preparing gold probes for multiple-labeling cytochemistry European Journal of Cell Biology 1985 38 87 93 4029177

[B66] Wu J Zhang X Nie Y Luo X High-efficiency transformation of Gossypium hirsutum embryogenic calli mediated by *Agrobacterium tumefaciens *and regeneration of insect-resistant plants Plant Breeding 2005 124 142 146 10.1111/j.1439-0523.2004.01056.x

[B67] Amano T Hirasawa K O'Donohue MJ Pernolle JC Shioi Y A versatile assay for the accurate, time-resolved determination of cellular viability Analytical Biochemistry 2003 1 7 10.1016/S0003-2697(02)00653-X 12633596

[B68] Lipka V Dittgen J Bednarek P Bhat R Wiermer M Stein M Landtag J Brandt W Rosahl S Scheel D Francisco LF Molina A Parker J Somerville S Schulze-Lefert P Pre- and postinvasion defenses both contribute to nonhost resistance in Arabidopsis Science 2005 310 1180 1183 10.1126/science.1119409 16293760

[B69] Livak KJ Schmittgen TD Analysis of relative gene expression data using real-time quantitative PCR and the 2-CT method Methods 2001 25 402 408 10.1006/meth.2001.1262 11846609

