# Supplementary figures and images for: Genetic transformation of cotton with a harpin-encoding gene hpaXoo confers an enhanced defense response against different pathogens through a priming mechanism
Source: BMC Plant Biol. 2010 Apr 15;10:67. doi: 10.1186/1471-2229-10-67 (PMC3095341; doi:10.1186/1471-2229-10-67)

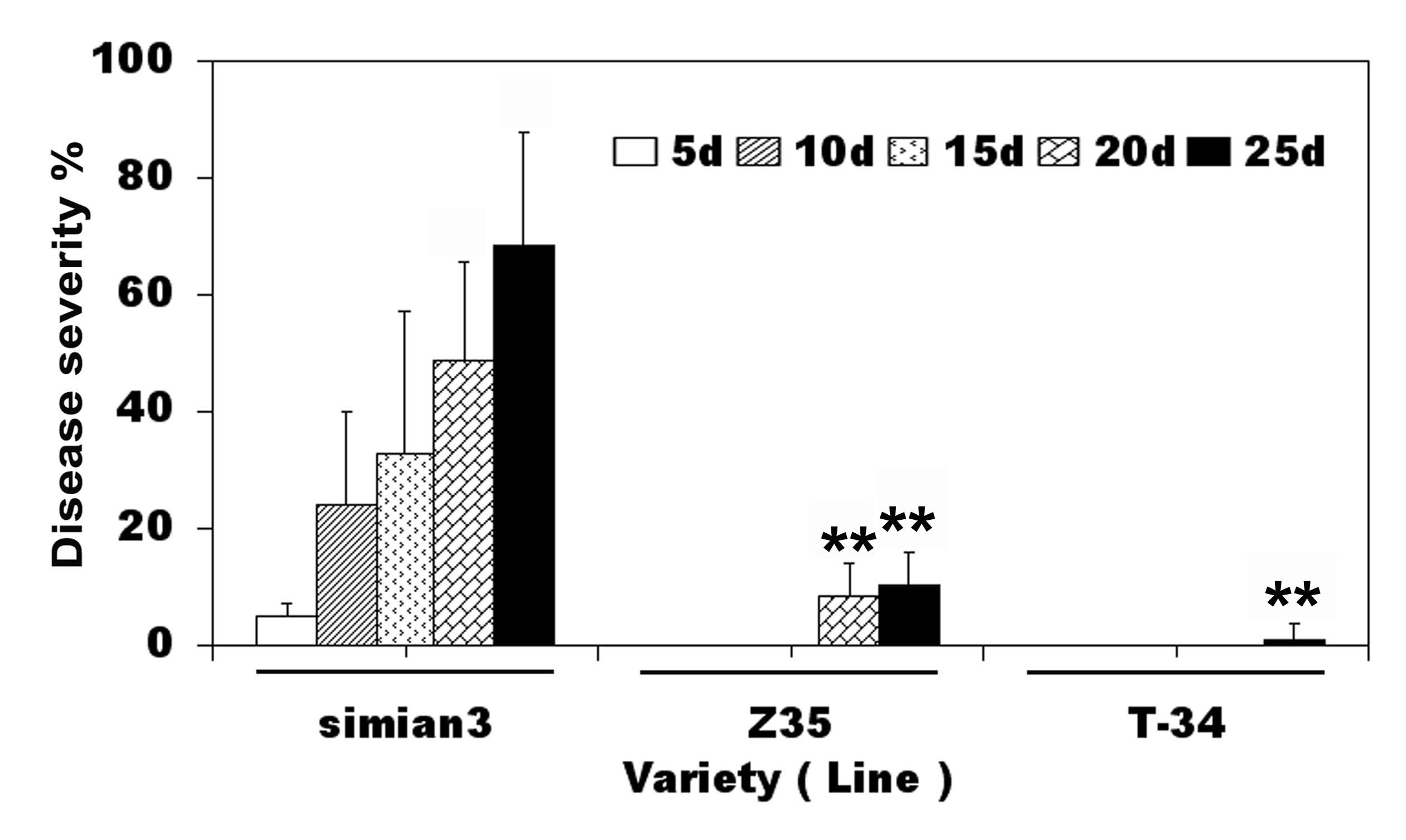

Supplement: Additional file 1 — Table S1. Stability of resistance to Verticillium dahliae in T1-T6 progenies of transgenic cotton line T-34. +: a score of 0-4 was given based on both external (foliar damage) and internal (vascular discoloration) symptoms 10 and 20 days after inoculation, respectively. Plants showed the ratings of 0 - 2 were counted as resistant (R) and those with the ratings of 3-4 were counted as susceptible (S). ++: +/- represented the presence/absence of the amplification product using hpa1Xoo specific primers in the PCR analysis. [file 1471-2229-10-67-S1.JPEG]

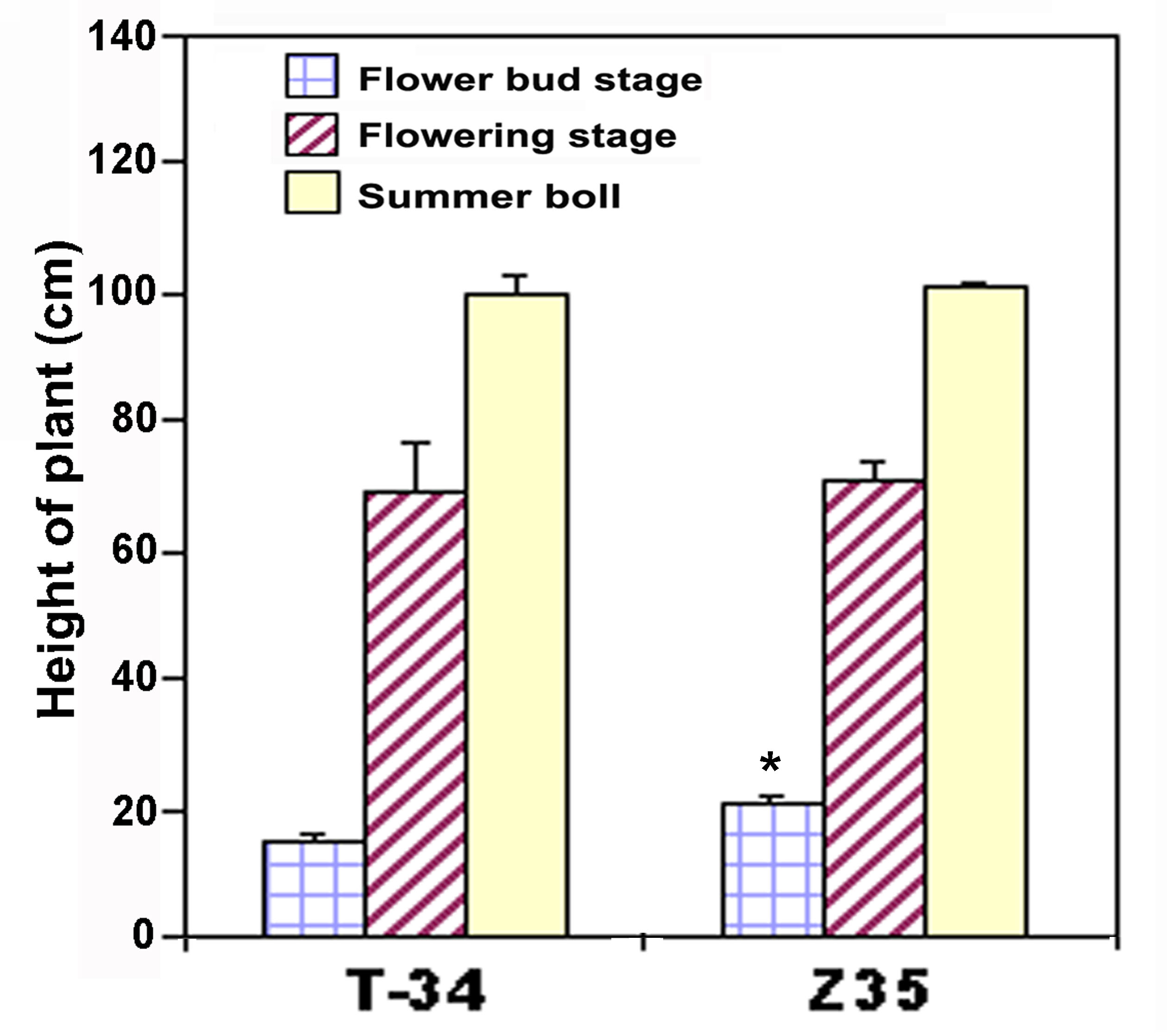

Supplement: Additional file 2 — Figure S1. The plant height of hpa1Xoo-transformed T-34 and untransformed Z35 at different growing stages. The experiment with three replications was performed at an independent field in Dafeng city, Jiangsu, CHINA. The fertilizer, irrigation, plant protection and other inter cultural practices were according to normal agronomic practices. The height of continued fifty plants was investigated each replication at same time. [file 1471-2229-10-67-S2.JPEG]
