# Supplementary material for: Genetic transformation of cotton with a harpin-encoding gene hpaXoo confers an enhanced defense response against different pathogens through a priming mechanism
Source: BMC Plant Biol. 2010 Apr 15;10:67. doi: 10.1186/1471-2229-10-67 (PMC3095341; doi:10.1186/1471-2229-10-67)
Supplement: Additional file 3 — Figure S2. Isolate F24 of Fusarium oxysporum f. sp. vasinfectum (FOV) race 7 was used in this study. Inoculum was prepared by autoclaving cotton seed (at 121°C and 103.4 kPa for 20 min) twice and mixing it with monoconidial cultures of Fov that had been grown on PDA. When fully colonized (10 days), the inoculum was mixed with pasteurized UC potting mix (sorghum: potting mix, 1:10 v/v) in plastic bags and incubated for 4 weeks. The colonized cotton seed-UC mix was then added to more pasteurized potting mix (1:1, v/v) and distributed equally into pots 9 cm in diameter. The transgenic hpa1Xoo cotton line T-34, the receptor Z35, and the susceptible cotton cultivar Simian 3 were grown from seed in the potting mix containing the inoculum. One plant of each cultivar was grown in each pot and there were 10 replications (pots) of each treatment (isolate of Fov). The experiments were repeated three times. All plants were grown under 12 h of light at 24-29°C and 70%-90% relative humidity. Individual plants were rated for disease severity based on the following scale for vascular discoloration. The discoloration was scored (y) for every internode. 0 = no vascular staining evident, 1 = light vascular staining evident as spotty areas, 2 = more contiguous staining covering an area equal to between one-quarter and one-half of the transverse section of the stem, 3 = moderate vascular staining (intensity of the brown/black color) evident as a band extending over nearly all of the transverse section, 4 = vascular staining darker or the plant dead. The disease index (DI) was calculated as follows: DI = 100∑y/4d, where (d) is the total number of seedling internodes including hypocotyls and (4) is the maximum score for an internode. Mean values of DI were calculated based on four replicates for both inoculated and control plants. Asterisks represent significant differences at the level of 0.01. [file 1471-2229-10-67-S3.DOC]

**Table S1** Stability of resistance to *Verticillium dahliae* in T1-T6 progenies of transgenic cotton line T-34

| Generation | Plants assayed | Resistance to *Verticillium dahliae* | | Total plants tested by PCR | Number of plants | Presence of *hpa1Xoo^++^* |
| --- | --- | --- | --- | --- | --- | --- |
|  |  | Resistance | Plants |  |  |  |
| T1 | 50 | R^+^ | 12 | 30 | 7 | + |
|  |  | S | 38 |  | 23 | - |
| T2 | 57 | R | 32 | 30 | 24 | + |
|  |  | S | 25 |  | 6 | - |
| T3 | 68 | R | 53 | 30 | 23 | + |
|  |  | S | 15 |  | 7 | - |
| T4 | 83 | R | 62 | 30 | 22 | + |
|  |  | S | 21 |  | 8 | - |
| T5 | 75 | R | 57 | 30 | 24 | + |
|  |  | S | 18 |  | 6 | - |
| T6 | 71 | R | 53 | 30 | 24 | + |
|  |  | S | 18 |  | 6 | - |

^+^: a score of 0-4 was given based on both external (foliar damage) and internal (vascular discoloration) symptoms 10 and 20 days after inoculation, respectively. Plants showed the ratings of 0 – 2 were counted as resistant (R) and those with the ratings of 3-4 were counted as susceptible (S).

^++^: +/- represented the presence/absence of the amplification product using *hpa1Xoo* specific primers in the PCR analysis.
